# Non-contrast enhanced functional lung MRI in children: report on 900 own measurements using matrix-pencil decomposition (MP-) MRI

**DOI:** 10.3389/fped.2025.1519148

**Published:** 2025-03-13

**Authors:** Carmen Streibel, Grzegorz Bauman, Oliver Bieri, Orso Pusterla, Enno Stranzinger, C. Corin Willers, Marion Curdy, Carmen Casaulta, Bettina Sarah Frauchiger, Insa Korten, Sophie Yammine, Yasmin Salem, Philipp Latzin, Elisabeth Kieninger

**Affiliations:** ^1^ Division of Paediatric Respiratory Medicine and Allergology, Department of Paediatrics, Inselspital, Bern University Hospital, University of Bern, Bern, Switzerland; ^2^ Graduate School for Health Sciences, University of Bern, Bern, Switzerland; ^3^ Department of General Internal Medicine and Psychosomatic, Heidelberg University Hospital, University of Heidelberg, Heidelberg, Germany; ^4^ Department of Radiology, Division of Radiological Physics, University of Basel Hospital, Basel, Switzerland; ^5^ Department of Biomedical Engineering, University of Basel, Allschwil, Switzerland; ^6^ Department of Diagnostic and Interventional and Pediatric Radiology, Inselspital, Bern University Hospital, Bern, Switzerland

**Keywords:** functional lung MRI, children, lung function, MP-MRI, pulmonology

## Abstract

**Objectives:**

Functional imaging of the lungs enables a spatially resolved examination of pulmonary ventilation and perfusion. Non-contrast-enhanced (NCE) magnetic resonance imaging (MRI) techniques do not require specialized set-ups (e.g., hyperpolarized gases), but are applicable on standard clinical MRI scanners. Since patients are not exposed to ionizing radiation during the examinations, NCE-MRI is highly attractive for use in pediatrics, especially in children with chronic lung diseases requiring repeated follow-up measurements.

**Study design:**

We report on our own experience of more than 900 NCE-MRI measurements in children over seven years using matrix pencil decomposition (MP-)MRI. We present original data, i.e., clinical cases in which MP-MRI helped in clinical decision-making together with valuable practical points.

**Results:**

At our center, an optimized workflow including a child-friendly setting and automated provision of outcome protocols led to great acceptance of functional NCE-MRI in patients and clinicians. Within this setting, regular MP-MRI measurements were successfully implemented into clinical routine and proved to be very helpful for surveillance and specific clinical decision-making. We present exemplary cases illustrating the potential of NCE-MRI as a diagnostic tool.

**Conclusion:**

In this article, we summarize our unique experience of a large number of MP-MRI measurements. We give an overview on our workflow including standardized and automated analysis and reporting. The exemplary cases from different disease groups illustrate its value in the clinical setting. In conclusion, visualizing regional functional deficits and respective underlying pathophysiological nature of lung impairment seems promising for increasing use of NCE-MRI in the future.

## 1 Introduction

Recent technical developments have not only enabled the acquisition of high-quality morphological images of the lungs using magnetic resonance imaging (MRI) (1–4), but also the implementation of functional lung MRI (1, 2, 5). Functional MRI examinations provide spatially resolved information on lung ventilation or perfusion (1, 2, 5). While some approaches require either the inhalation of hyperpolarized gases (2) or the use of intravenous contrast agents (1), so-called non-contrast-enhanced (NCE) functional MRI techniques and the respective scan sequences are applicable on standard clinical MRI scanners without the need of specialized set-ups (6–16). A voxel-wise analysis of signal changes over time within an image series enables the detection of local ventilation or perfusion deficits (6–16). Because of its easy application and since patients are not exposed to ionizing radiation during the examinations, NCE-MRI is highly attractive for use in pediatrics, especially in children with chronic lung diseases requiring repeated follow-up measurements. The available NCE-MRI techniques include matrix-pencil decomposition (MP-) MRI, where an highly optimized signal-to-noise ratio in the lung tissue is achieved by exploiting an ultra-fast balanced steady-state free precession (uf-bSSFP) pulse sequence for data acquisition (7, 17, 18). MP-MRI is in use in our center since 2017, resulting in a large number of examinations in children. We are therefore able to share valuable key experiences and exemplary clinical cases to the broad readership including clinicians interested in implementing and applying this innovative technique.

In the first part of this article, we summarize our own experience of more than 900 MP-MRI measurements in children. In the second part, we present exemplary cases of MP-MRI in the clinical setting.

## 2 More than 900 MP-MRI measurements in children—report on our own experience

**Figure 1:**
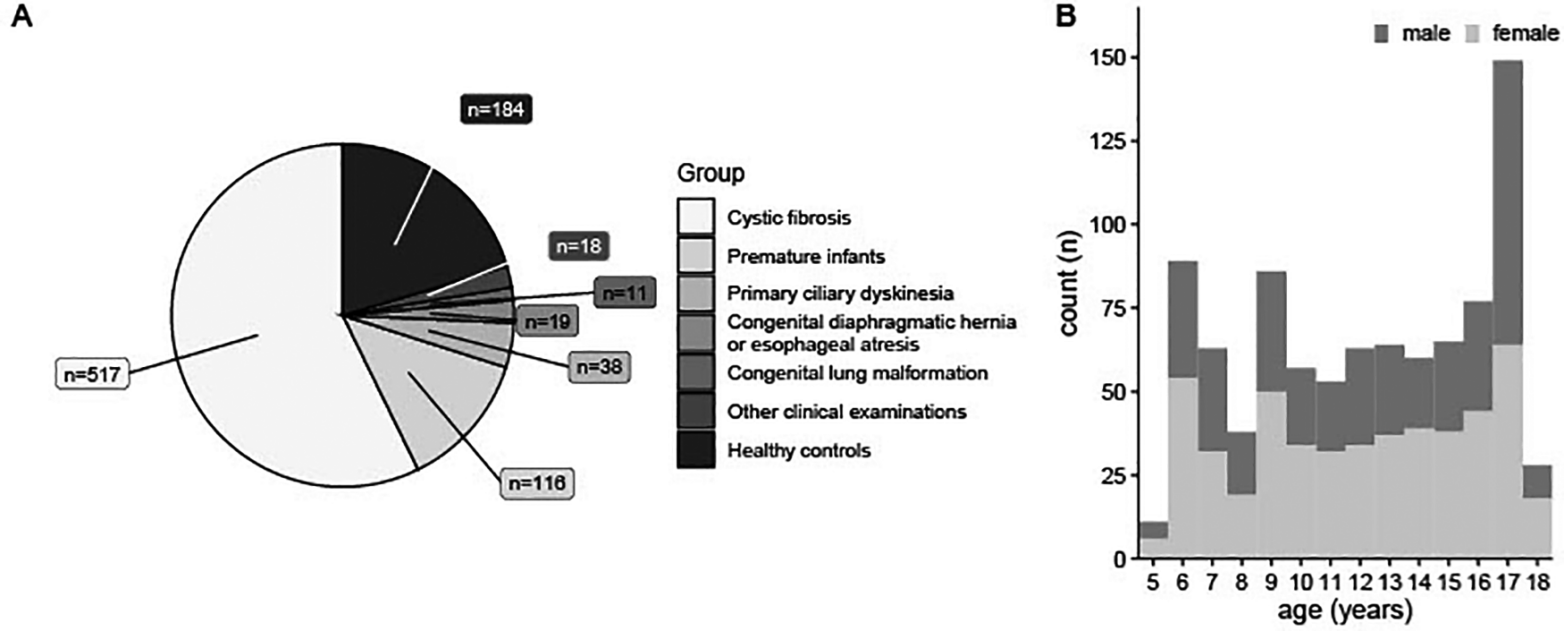
903 MP-MRI measurements in 473 individuals during seven years. **(A)** MP-MRI measurements sorted by disease groups. **(B)** Age-Distribution of patients having performed MP-MRI measurements, sorted by sex. *n* (absolute number of measurements). MRI, magnetic resonance imaging; MP-MRI, matrix pencil decomposition MRI.

At the Children's University Hospital of Bern, we have been using MP-MRI since November 2017. We have performed more than 900 functional lung MRI scans in 473 children aged between 5 and 18 years. Since MP-MRI is part of most our study protocols, data presented are from cohort studies, (Bern Infant Lung Development (BILD) cohort (19), Swiss Cystic Fibrosis Infant Lung Development (SCILD) cohort (20)), clinical studies and scientific exploration of complex clinical cases. Figure 1 presents the disease groups covered and the age distribution of the children included.

**Figure 2:**
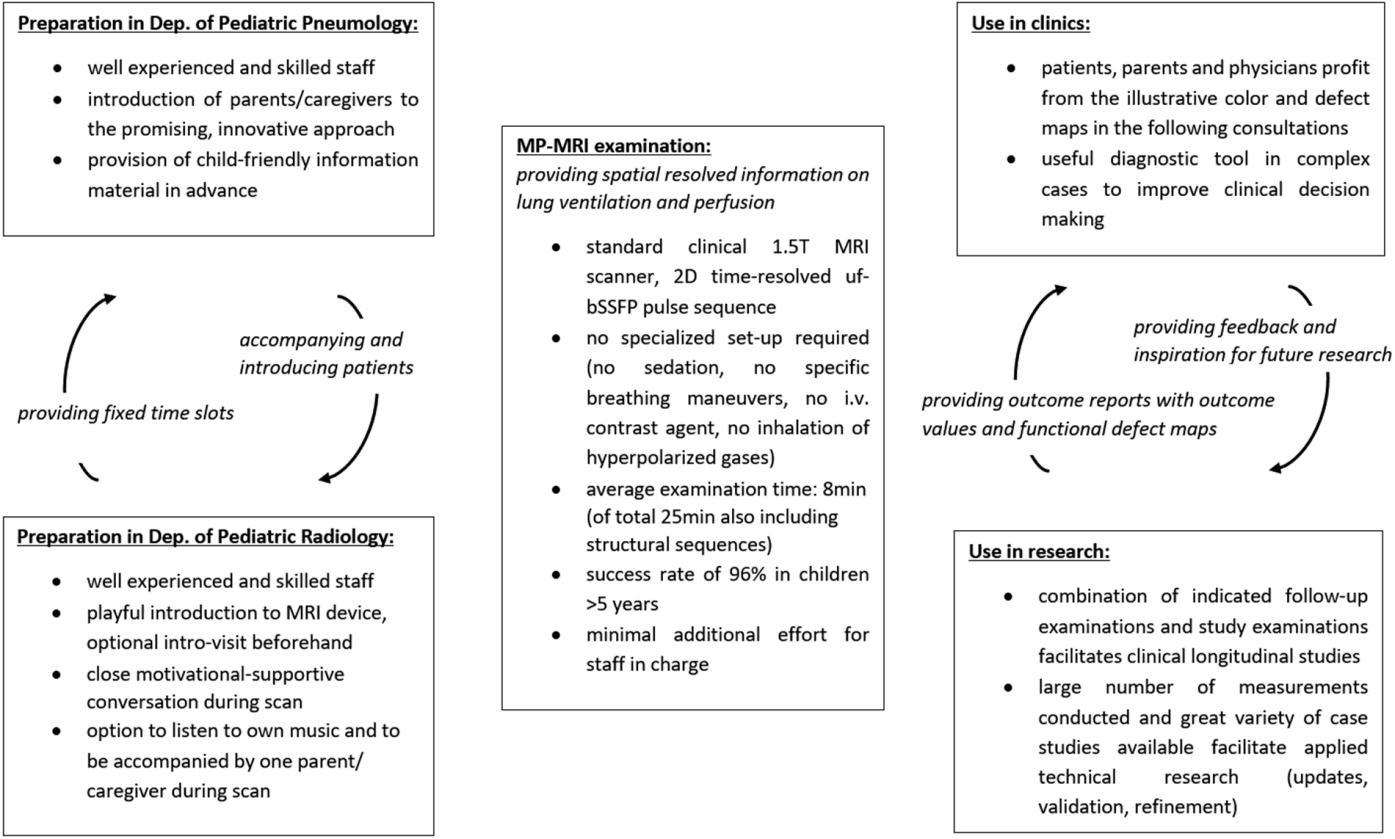
Overview on the applied workflow having ensured successful implementation of MP-MRI measurements. MRI, magnetic resonance imaging; Dep., department; min, minutes; MP-MRI, matrix pencil decomposition MRI; T, Tesla; 2D, two dimensional.

From our experience, we will herein report on specific key points and prerequisites for a successful implementation (Figure 2).

### 2.1 Preparation and child-friendly setting

As generally known, it proved to be particularly helpful to prepare the children and their parents/caregivers in advance for the upcoming MP-MRI examination.

**Figure 3:**
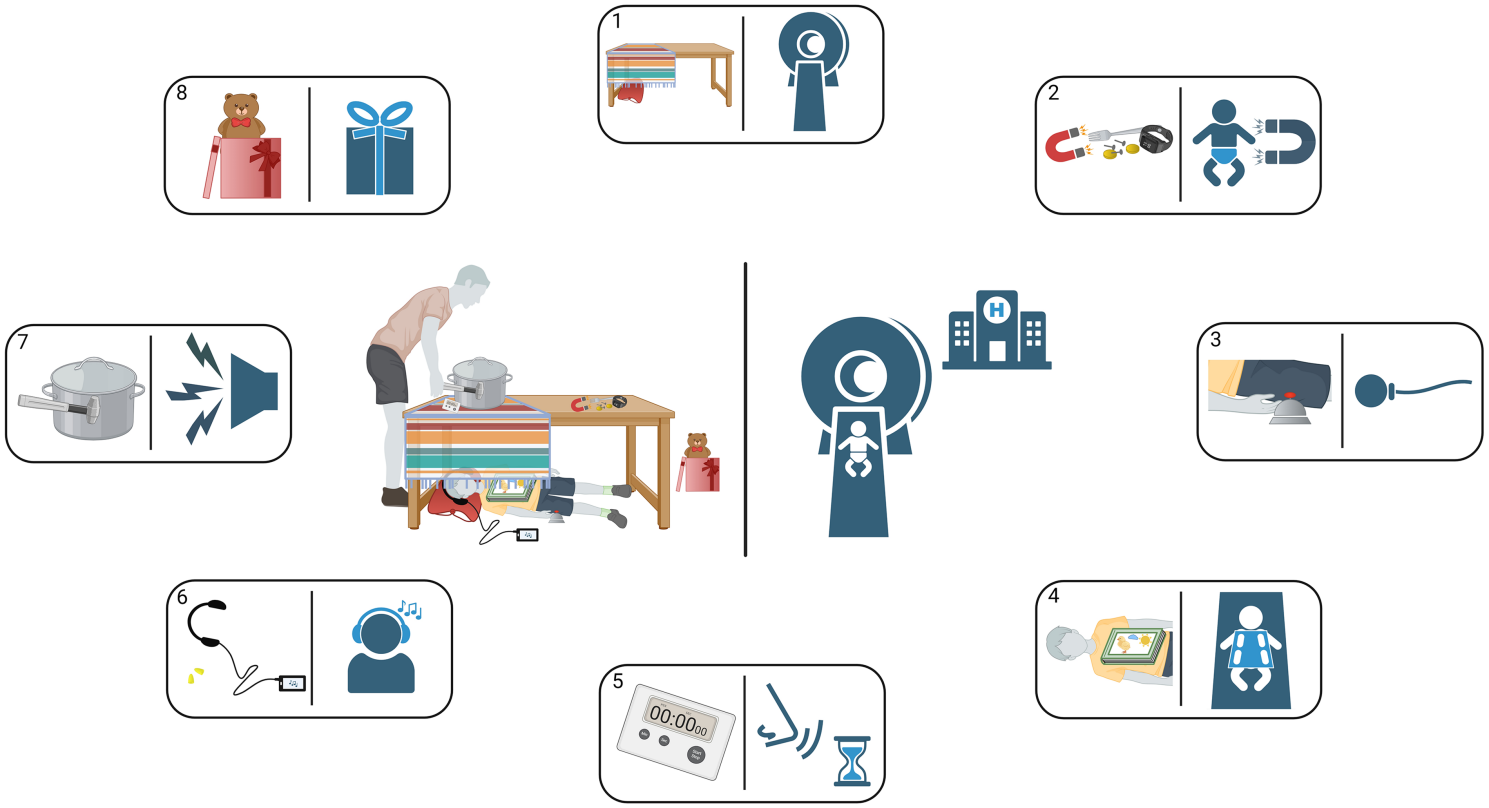
Instructions for a playful introduction to an upcoming MP-MRI scan at home—illustration. In preparation for the upcoming MP-MRI measurement, the children and their parents or care givers receive this illustration together with instructions from Table 1 so that they can familiarize themselves with the expected conditions already at home. The numbers in the rectangles refer to the corresponding rows in Table 1.

The following protocol for the MRI examinations has been implemented: (i) distribution of child-friendly information material including imitation of MRI measurement at home (Figure 3 and Table 1), (ii) invitation to an intro-visit at the MRI, (iii) age-appropriate, playful introduction to the MRI device on-site (e.g., fairy tale about resident little magnet wizards, practical experiment using a tennis ball filled with nails etc.), (iv) opportunity to listen to self-chosen audio plays or music during the scan, (v) parents can stay in the scanner cabin and (vi) close motivational-supportive conversation with the child during the examination via intercom. With this approach, we achieved a feasibility rate of 96% from 5 years age onwards (number of completed scanning protocol and diagnostic image quality suitable for post-processing divided by number of attempted scans, shown in a prospective, observational study) (21). Of 52 children aged between 5 and 7 years, 50 children completed the full scanning protocol, one child refused lying in the scanner and another child stopped the examination after one acceptable functional scan resulting in an uncompleted morphological scan. The most decisive factor for good feasibility was age as in children aged between 3 and 4.9 years only 10 of 30 individuals agreed on lying in the scan device (feasibility rate 33%). However, these 10 patients, willing to start the examination, completed it successfully (21).

**Table 1:** Instructions for a playful introduction to an upcoming MP-MRI scan at home—checklist. Achieved steps can be rewarded using a sticker or a stamp.

Step	You don't need to be worried about the upcoming MRI scan—you can play out what's to come at home together with one of your parents!	Achieved?
1	Build your own small MRI scanner at home. Take a table that is big enough for you to lie under and cover one half with a cloth or blanket hanging over the sides. When you lie down under it later, it will feel a bit like a cave for your head and upper body. Just like later in the MRI device. Don't forget to keep a pillow ready to make it cozy!	
2	There are very large and strong magnets in the MRI scanner. Do you know which objects are all attracted to them? Take a small magnet you have at home and look for suitable objects in your surroundings, e.g., coins, nails, cutlery. Then test whether there are any metal objects on your clothes (trouser button, watch, jewelry). For the measurement, you need to put on clothes without the corresponding properties, e.g., jogging pants, and take off other accessories. Right now, it is best to place them on the table.	
3	During the measurement, the nurse in charge will not be in the same room as you, but will be able to see you through a window and will always be available if you need her/him. You will be given a small bell (rubber ball) to press so that you can contact them. They will then hear you ring like a telephone, ask you what's up and you can tell your concern. Find a small bell or squeaky cuddly toy in the house now and put it under the table for later.	
4	To make the MRI images of your lungs even more precise, a so-called surface coil will be placed on your chest during the measurement. This surface coil looks similar to a plastic swimming board. Get used to the feeling by looking for a large book, not too heavy, which you can later place on your chest. Imagine it's like a large cell phone or tablet that takes photos of your lungs.	
5	It is important that your hearing is protected from the noise of the MRI scanner during the measurement. You will therefore be given earplugs. Do you have any at home? You will also be allowed to listen to your own favorite music or radio play via headphones during the scan. So set your own favorite song on your cell phone or CD player and headphones!	
6	Are you ready? Great! Then you can crawl under the table, put your head on the pillow and make yourself comfortable. Are the earplugs in and the headphones on? Get the bell ready so that you can use it. Then place the book on your chest. The measurement can start! At the very beginning, you need to hold your breath for about 10 s to calibrate the device. Have your parent stop the time. Can you do it?	
7	The measurement is running … Can you manage to lie very still, like a statue? Be careful, during the measurement the MRI machine sometimes rattles quite loudly, almost like on a building site. That doesn't have sto scare you. You can get used to it at home by having your parent make some noise on the table. A saucepan to bang against is quite suitable, or a musical instrument or something similar. During this scan simulation, you might also try out the bell or squeaker you have prepared.	
8	Congratulations, you've made it! For sure, your parents are really proud of you that the measurement went so smoothly. You have well deserved a little surprise waiting for you… Does your parent dare to lie in your little MRI scanner while you make the noise? Feel free to swap roles. So that nobody feels alone during the measurement, you can also show each other that you are there by touching/stroking your feet. This will also be allowed during the measurement (an accompanying person is permitted in the measurement room).	

In the following medical consultations, both patients and physicians profit from the MP-MRI color-coded and defect maps, visualizing affected regions and extent of ventilation and perfusion impairment.

### 2.2 Technical set-up

In our center, MP-MRI measurements are performed on a clinical whole-body Siemens 1.5 T scanner (applicability on scanners of other manufacturers and corresponding adaptation of the sequences is currently in progress). A close collaboration between Pediatric Pulmonology and Pediatric Radiology as well as fixed time slots per week facilitate applicability. We apply functional alongside structural sequences to ensure a comprehensive imaging of the lung, with MP-MRI accounting for 7.9 + −1.8 min of the average examination time of 25.1 + −4.6 min (21). To avoid longer investigations than usual, we limited the structural sequences to the most relevant ones (details presented in the OLS). As no sedation or specific breathing maneuvers are required, the additional effort for staff in charge is minimal. MP-MRI sequences are done at the end and as such, only measurement zones need to be defined.

### 2.3 Analysis and outcome calculation

The analysis of the MP-MRI scan data obtained and the computation of outcome values is currently carried out using a pipeline In our center, this is sustained by specialized biomedical engineer. But as the current version of the processing pipeline is available as a docker container, there is no strict qualification requirement. The software can be also installed and maintained by an IT person with knowledge of a Linux operating system on an intermediate level. However, the software is still in a translational phase and is not a commercially certified product. Thus, there is no dedicated official support for it.

We have successfully automated each step for reproducible application in research and clinics. The total calculation time required is 20 min per subject (22). Regarding eventual motions of the young patients during the MRI examination, the amplitude of motion is detected during the image registration process. Images with a motion amplitude higher than a threshold will be discarded in the further analysis. However, as with every MRI examination, a severe bulk motion during the scan will result with inferior or undiagnostic image quality. Further, we implemented a validated artificial neural network to automatically segment the lung areas on the MRI images (23) and the subsequent analysis are conceptually designed to be robust to minor segmentation inaccuracies (22). Further, we replaced time-consuming visual scoring of defect maps (24) by quantifiable and automatically calculable outcome values: (i) relative lung volume with impaired ventilation or perfusion (VDP, QDP) (21, 23, 25–31), (ii) relative lung volume with combined ventilation and perfusion defect (VQD_match_) (30) and (iii) defect homogeneity (defect distribution index: DDI) (32). The latter numerically quantifies how clustered or scattered the impaired lung regions are which would otherwise have to be recognized visually by the human observer (32). Finally, an automatically generated pdf report includes color-coded maps in addition to numerical values (Supplementary Figure S2).

Additionally, we constantly incorporate the user feedback from clinicians, radiologists and research group members in close cooperation with computer scientists and physicists involved. When investigating innovative ideas and implementing updates, the immense number of measurements conducted and the great variety of case studies available have proven to be central to success.

### 2.4 Conclusion

We report here the positive experiences with the implementation of MP-MRI measurements in our department. Especially the non-invasiveness of the examination contributes to the acceptance of the examination in healthy children within study protocols. The children quickly lose their fear of the MRI device if they are gently introduced to it in advance. As MP-MRI is done within few minutes, adding the sequences to structural sequences does not change overall scan time substantially. The most challenging point in our institution was the installation of the sequences on the different MRI devices along with training of staff and connecting the data analysis pipeline for an automated workflow with the clinical information system, as the NCE-MRI techniques are not FDA-approved. However, the achieved unique collection of follow-up measurements and case studies has been a great resource for configuring reliable updates and exploring innovative ideas.

## 3 Clinical cases

In the following section, we report on exemplary clinical cases in which MP-MRI was of great use not only as outcome parameter and part of the study setting, but also as very helpful diagnostic tool for specific clinical decision-making. For better readability, detailed numerical outcome values for each case are presented in Table 2.

**Table 2:** Detailed MP-MRI and lung function outcome data for the case studies presented in Figure 4 to Figure 8.

Case 1 I.	Overall VDP 28.7%, overall QDP 28.3%, overall VQD_match_ 16.6%, DDI_V_ 3.1, DDI_Q_ 3.4, FEV_1_ z-score −2.2, LCI_2.5_ 10.3
Case 1 II.	Overall VDP 21.6%, overall QDP 25.2%, overall VQD_match_ 4.6%, DDI_V_ 0.6, DDI_Q_ 2.2, FEV_1_ z-score 0.5, LCI_2.5_ 7.6
Case 1 III.	Overall VDP 21.7%, overall QDP 18.5%, overall VQD_match_ 1.9%, DDI_V_ 0.5, DDI_Q_ 0.4, FEV_1_ z-score 0.2, LCI_2.5_ 6.7
Case 1 IV.	Overall VDP 21.1%, overall QDP 18.8%, overall VQD_match_ 1.6%, DDI_V_ 0.3, DDI_Q_ 0.4, FEV_1_ z-score 0.6, LCI_2.5_ 6.6
Case 2 I.	VDP_right_ 19.6%, QDP_right_ 36.5%, VQD_match_right_ 10.7%, DDI_V_right_ 2.1, DDI_Q_right_ 11.3, FEV_1_ z-score −4.2, LCI_2.5_ 6.8
Case 2 II.	VDP_right_ 31.1%, QDP_right_ 37.8%, VQD_match_right_ 14.5%, DDI_V_right_ 3.6, DDI_Q_right_ 9.3, FEV_1_ z-score −3.1, LCI_2.5_ 7.6
Case 2 III.	VDP_right_ 32.1%, QDP_right_ 32.4%, VQD_match_right_ 13.8%, DDI_V_right_ 3.4, DDI_Q_right_ 6.1, FEV_1_ z-score −3.1
Case 3	Overall VDP 21.8%, overall QDP 23.7%, overall VQD_match_ 6.5%, DDI_V_ 0.6, DDI_Q_ 1.0, FEV_1_ z-score −2.5, FVC z-score −1.6, LCI_2.5_ 10.7
Case 4	VDP_right_ 40.8%, QDP_right_ 38.6%, VQD_match_right_ 17.8%, DDI_V_right_ 2.1, DDI_Q_right_ 3.7, FEV_1_ z-score −2.2, FEV_1_/FVC z-score −2.3, RV/TLC_pp_ 35.6, LCI_2.5_ 7.7
Case 5	VDP_left_ 30.6%, QDP_left_ 55.0%, VQD_match_left_ 21.7%, DDI_V_left_ 3.8, DDI_Q_left_ 6.0, VDP_right_ 17.6%, QDP_right_ 5.2%, VQD_match_right_ 0.3%, DDI_V_right_ 5.8, DDI_Q_right_ 1.3, FEV_1_ z-score −3.5, FEV_1_/FVC z-score −2.6, RV/TLC_pp_ 41.4, LCI_2.5_ 6.8

VDP, ventilation defect percentage; QDP, perfusion defect percentage; VQD_match_, combined/matched ventilation and perfusion defect; DDI, defect distribution index; Q, perfusion; V, ventilation; FEV_1_, forced expiratory volume in 1 s; FVC, functional vital capacity; RV, residual volume; TLC, total lung capacity; pp, percent predicted; LCI_2.5_, lung clearance index measured at 2.5% of the normalized nitrogen starting concentration.

### 3.1 Case 1: patient with cystic fibrosis (CF)

**Figure 4:**
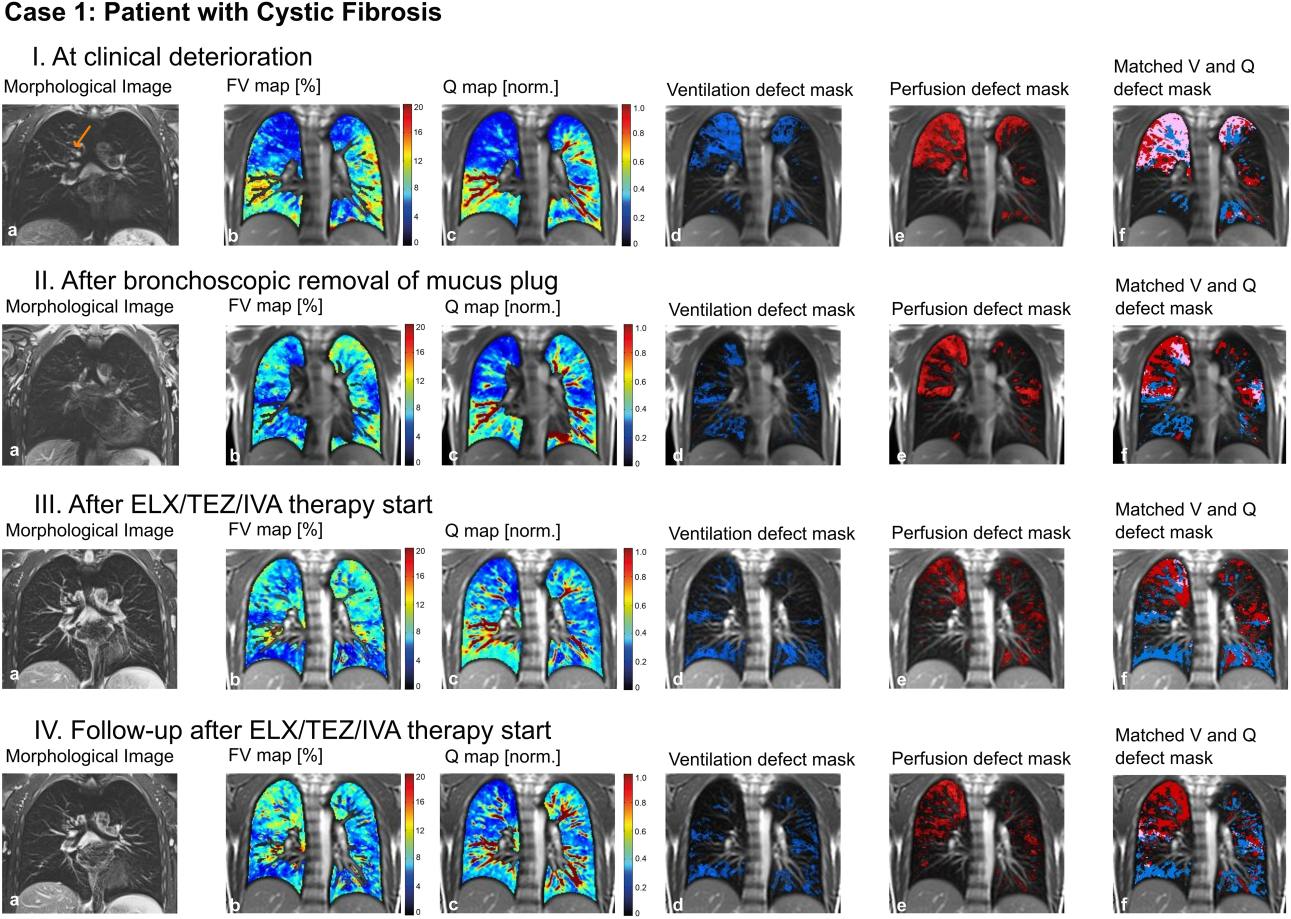
Case 1. 14-year-old patient with cystic fibrosis (I) at clinical deterioration including mucus plugging of the right upper lobar bronchus (arrow), (II) after bronchoscopic removal of mucus plug, (III) after ELX/TEZ/IVA therapy start and (IV) at follow-up after ELX/TEZ/IVA therapy start. Morphological images (coronal T2 blade FS sequence, 5 mm) **(a)**, fractional ventilation **(b)** and perfusion **(c)** maps, masks representing areas with impaired ventilation **(d)**, perfusion **(e)** and matched ventilation and perfusion defects **(f)** are shown. On the heat maps, a change of color range towards dark blue indicates severe impairment of lung ventilation or perfusion. ELX/TEZ/IVA, elexacaftor/tezacaftor/ivacaftor combination regimen; FV, fractional ventilation; MP-MRI, matrix-pencil magnetic resonance imaging; Q, perfusion; V, ventilation.

This is a 14-year-old male patient with CF before the approval of triple cystic fibrosis transmembrane conductance regulator (CFTR)-modulator therapy. He showed clinical deterioration and lung function decline over the last months. We performed an MP-MRI scan (Figure 4) in addition to structural MRI. The pronounced impairment of ventilation and perfusion in the right upper lung shown in MPI-MRI was together with the morphological images suggestive of mucus plugging of the right upper lobar bronchus. After bronchoscopic removal of the large mucus plug, symptoms resolved. Lung function improved and a follow-up MP-MRI revealed improvement of lung ventilation and perfusion at the right upper lobe. A sustained recovery of lung function was achieved later through introduction of CFTR modulator therapy.

To conclude: MP-MRI helped to identify and quantify the region of poorly ventilated and/or perfused areas, that potentially were responsive to bronchoscopic intervention.

### 3.2 Case 2: patient with necrotizing pneumonia

**Figure 5:**
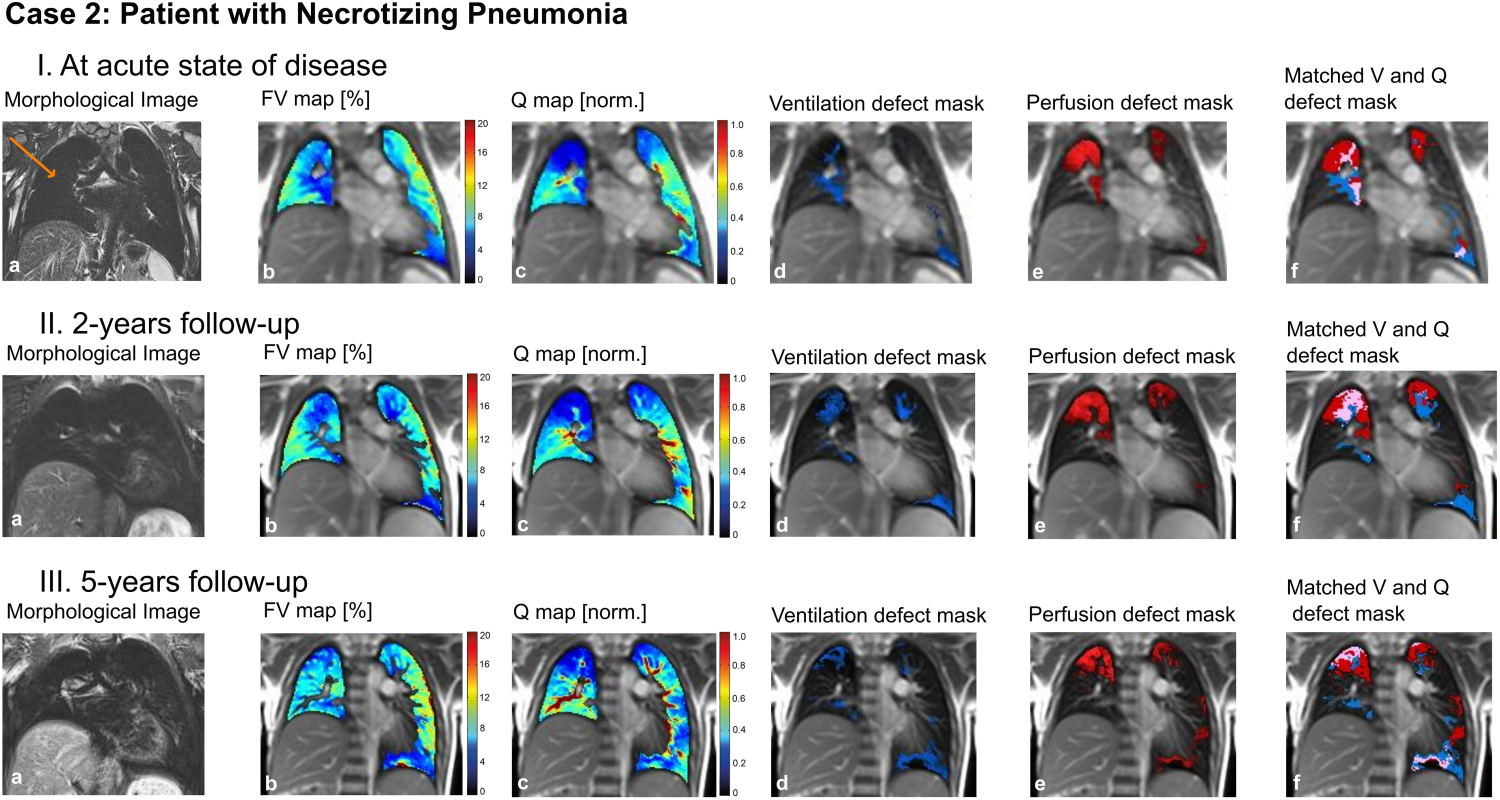
Case 2. 5-year-old patient with necrotizing pneumonia, surgical removal of the right upper lung lobe and remaining right middle and lower lung lobe (arrow) (I) at acute state of disease, (II) at 2-years follow-up and (III) at 5-years follow-up. Morphological images (coronal T2 blade FS sequence, 5 mm) **(a)**, fractional ventilation **(b)** and perfusion **(c)** maps, masks representing areas with impaired ventilation **(d)**, perfusion **(e)** and matched ventilation and perfusion defects **(f)** are shown. On the heat maps, a change of color range towards dark blue indicates severe impairment of lung ventilation or perfusion. FV, fractional ventilation; MP-MRI, matrix-pencil magnetic resonance imaging; Q, perfusion; V, ventilation.

In a 5-year-old boy, necrotizing pneumonia of the right lung resulted in surgical removal of the right upper lobe. A prolonged course of disease with significant respiratory limitations and persistent oxygen requirement followed. A computed tomography (CT) scan (not shown) revealed marked dystelectatic and scarring changes of the remaining right lung. To address the discussion whether this specific part of the lung was still functional (thus ventilated and perfused) or should also be removed in order to reduce the risk of further infections, we performed an MP-MRI measurement (Figure 5). The scan showed impaired, but existing perfusion and adequate ventilation of the remaining lung indicating residual function. Consequently, the resection was waived. In the further course, the patient improved clinically with improvement of lung function. A follow-up MP-MRI two and four years later showed normal ventilation and only slightly impaired perfusion in the lung area affected.

To conclude: MP-MRI allowed to specifically examine the post-infectious function of a lung lobe and helped in the discussion of indication for surgical intervention.

### 3.3 Case 3: patient with sickle-cell disease

**Figure 6:**
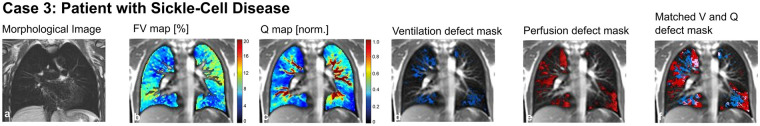
Case 3. 16-year-old patient with sickle-cell disease. Morphological images (coronal T2 blade FS sequence, 5 mm) **(a)**, fractional ventilation **(b)** and perfusion **(c)** maps, masks representing areas with impaired ventilation **(d)**, perfusion **(e)** and matched ventilation and perfusion defects **(f)** are shown. On the heat maps, a change of color range towards dark blue indicates severe impairment of lung ventilation or perfusion. FV, fractional ventilation; MP-MRI, matrix-pencil magnetic resonance imaging; Q, perfusion; V, ventilation.

A 16-year-old male adolescent with sickle-cell disease (SCD) suffered from recurrent episodes of acute chest syndrome. Lung function showed a mixed ventilatory impairment with airway obstruction, reduced functional vital capacity and increased ventilation inhomogeneity. For a better understanding of the underlying pathophysiological aspects, we performed an MP-MRI scan (Figure 6). We found normal ventilation but patchy-dispersed perfusion impairment, consistent with the vaso-occlusive nature of sickle cells.

To conclude: MP-MRI helped to visualize the pathophysiological nature of lung impairment, i.e., vasoocclusive processes, and to rule out other potential causes of lung pathology in this patient with SCD.

### 3.4 Case 4: patient with bronchiolitis obliterans

**Figure 7:**
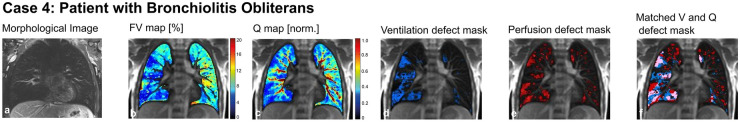
Case 4. 14-year-old patient with bronchiolitis obliterans. Morphological images (coronal T2 blade FS sequence, 5 mm) **(a)**, fractional ventilation **(b)** and perfusion **(c)** maps, masks representing areas with impaired ventilation **(d)**, perfusion **(e)** and matched ventilation and perfusion defects **(f)** are shown. On the heat maps, a change of color range towards dark blue indicates severe impairment of lung ventilation or perfusion. FV, fractional ventilation; MP-MRI, matrix-pencil magnetic resonance imaging; Q, perfusion; V, ventilation.

This is a 14-year-old male patient with post-infectious bronchiolitis obliterans. Diagnostic tests showed fixed airway obstruction and hyperinflation, regular ventilation inhomogeneity and typical radiological findings in CT scans (mosaic perfusion, bronchiectasis of the right upper and lower lung lobes; images not shown). Using MP-MRI (Figure 7), we were able to link the information from global lung function derived from pulmonary function tests with spatially resolved functional impairment provided by the MP-MRI scans, illustrating that ventilation and perfusion were both impaired to a higher degree in the right lung compared to the left side.

To conclude: MP-MRI helps not only to characterize better the regional impairment of lung function in addition to global lung function tests measured at the mouth. In this patient with post-infectious bronchiolitis obliterans it helped to quantify functional impairment due to bronchiectasis and ventilation inhomogeneity and visualize this regional functional deficit for better understanding of the disease process.

### 3.5 Case 5: patient with congenital diaphragmatic hernia

**Figure 8:**
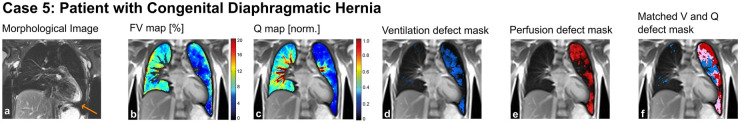
Case 5. 9-year-old patient with congenital diaphragmatic hernia (arrow) and post-natal repair using a muscle-flap. Morphological images (coronal T2 blade FS sequence, 5 mm) **(a)**, fractional ventilation **(b)** and perfusion **(c)** maps, masks representing areas with impaired ventilation **(d)**, perfusion **(e)** and matched ventilation and perfusion defects **(f)** are shown. On the heat maps, a change of color range towards dark blue indicates severe impairment of lung ventilation or perfusion. FV, fractional ventilation; MP-MRI, matrix-pencil magnetic resonance imaging; Q, perfusion; V, ventilation.

A 9-year-old girl with a large left-sided congenital diaphragmatic hernia (prenatal diagnosis and installation of an intratracheal plug, postnatal repair of the diaphragmatic hernia using a muscle flap) suffered from exercise-dependent respiratory symptoms. Lung function tests showed reduced vital capacity, hyperinflation and ventilation inhomogeneity. MP-MRI showed significantly decreased perfusion of the left lung combined with reduced ventilation (Figure 8). This clear picture was confirmed by the morphological MRI, where a rarification of the vessels was seen (panel a, Figure 8). This suggests altered fetal lung development and/or dysproportional catch-up growth [further results published in (30)].

To conclude: MP-MRI enables to examine and visualize the function of the left and right lung separately in patients with side-related anatomic aberrations.

### 3.6 Conclusion

In the clinical setting, different disease entities exist, in which patients, parents and care givers benefit from the opportunity to assess ventilation and perfusion of the lung in a spatially resolved manner and visualize any impairment. This helps in clinical decision-making regarding possible therapies, allows targeted follow-up of affected lung regions and helps to more specifically understand and explain the underlying lung physiology. The easy applicability and the non-invasiveness of MP-MRI examinations are additional factors in favor of their use.

## 4 Overall conclusion

The implementation of a standardized workflow in our center including automated analysis and reporting, was a pivotal step on the transition from use as research tool to regular clinical application. This has led to more than 900 MP-MRI examinations in the last years and quite some experience in its usefulness in different lung diseases. Based on this experience, we believe that MP-MRI is a promising tool for non-invasive assessment of ventilation and perfusion of the lung in diagnosis and surveillance of certain lung diseases. Comparison between centers and devices will be hopefully possible soon and allow even more widespread use.

## Data Availability

The raw data supporting the conclusions of this article will be made available by the authors, without undue reservation.
