# Supplementary material for: Non-contrast enhanced functional lung MRI in children: report on 900 own measurements using matrix-pencil decomposition (MP-) MRI
Source: Front Pediatr. 2025 Mar 13;13:1519148. doi: 10.3389/fped.2025.1519148 (PMC11947945; doi:10.3389/fped.2025.1519148)
Supplement: Supplementary file 1 [file Datasheet1.docx]

Online Supplement Material

**Non-contrast enhanced functional lung MRI in children: Report on 900 own measurements using matrix-pencil decomposition (MP-) MRI**

**Technical set-up: details on the structural MRI sequences applied**

**t2_hast_tra_5mm_mbh**

Generic name: **T2 HASTE** (Half Fourier Acquisition Single Shot Turbo Spin Echo)

Direction: Transverse (TRA)

Slice thickness: 5 mm

Additional information: MBH multiple breath hold

**t2_haste_cor_5mm_mbh**

Generic name: **T2 HASTE** (Half Fourier Acquisition Single Shot Turbo Spin Echo)

Direction: Coronal (COR)

Slice thickness: 5 mm

Additional information: MBH multiple breath hold

**UTE_fl3d_spiral_vibe_cor_1.25iso_5min30**

Generic name: **UTE (Ultrashort Echo Time)** 3D Spiral VIBE

Direction: Coronal (COR)

Voxel size: 1.25 mm isotropic

Additional information: This sequence combines UTE for capturing short T2* signals (useful for imaging bone, lungs, etc.), 3D Spiral acquisition for faster imaging. The sequence is breath-hold navigated in the expiration, length 5 min 30 sec.

**Supplemental Figure 1. Example of Matrix-Pencil MRI outcome report.**

| **Functional lung MRI report file** | | | |  | | |
| --- | --- | --- | --- | --- | --- | --- |
| Truelung version: 1.2 | | | | Evaluation date: 2024-01-01 00.00 | | |
| Method: AMP | | | | Thresholding: MEDIAN | | |
| **Patient data** | | | |  | | |
| Name: xxx  Patient ID: xxx  Birth date: xxx  Age: 13 y/o  Sex: M | | | |  | | |
| **Examination data** | | | |  | | |
| Station name: MR1 | | | | Examination date: 2024-01-01 00:00 | | |
| MR scanner: Aera | | | | Sequence: ufssfp2d1 | | |
| Baseline: syngo MR E11 | | | | Study ID: 0 | | |
| **Global outcomes** | | | |  | | |
| Function | SL | Vol [mL] | Defects [%] | VQO | Mean ± Std [Units] |  |
| Ventilation (V) | 7 | 1165 | 26.82 | 0.159 | 9.33 ± 5.14 |  |
| Perfusion (Q) | 7 | 1165 | 27.17 | 0.159 | 569.93 ± 300.96 |  |

# Lobar outcomes

| Function | Lobe | Vol [mL] | Defects [%] | Mean ± Std [Units] |
| --- | --- | --- | --- | --- |
| Ventilation (V) | LU | 271 | 21.73 | 11.37 ± 5.14 |
|  | LL | 237 | 12.86 | 8.99 ± 4.61 |
|  | RU | 249 | 53.58 | 7.19 ± 5.11 |
|  | RM | 112 | 23.41 | 11.85 ± 4.50 |
|  | RL | 292 | 21.36 | 8.55 ± 4.72 |
|  |  |  |  |  |
| Perfusion (Q) | LU | 271 | 18.56 | 587.35 ± 247.27 |
|  | LL | 237 | 18.21 | 651.97 ± 296.34 |
|  | RU | 249 | 66.24 | 340.36 ± 281.53 |
|  | RM | 112 | 8.33 | 698.63 ± 246.73 |
|  | RL | 292 | 16.34 | 633.50 ± 283.07 |

**Ventilation and perfusion maps**


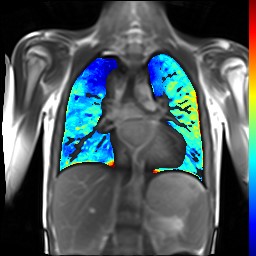

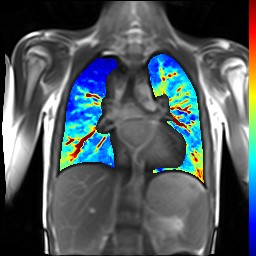

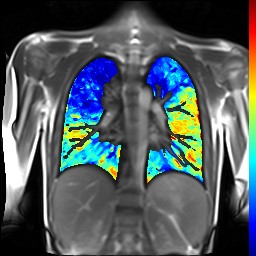

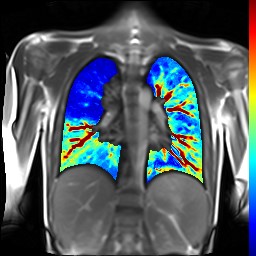

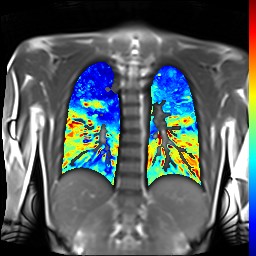

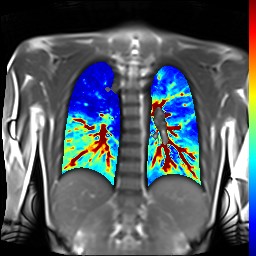

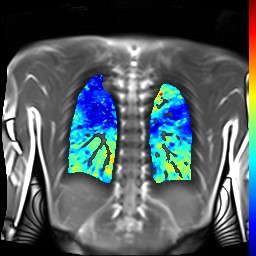

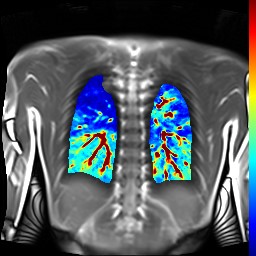


**Ventilation and perfusion impairment maps**


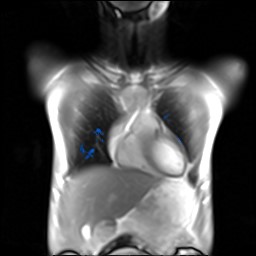

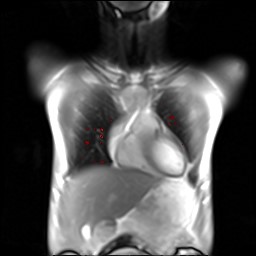

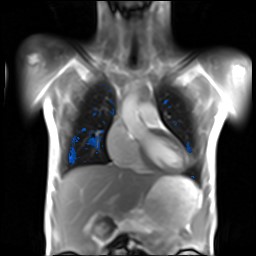

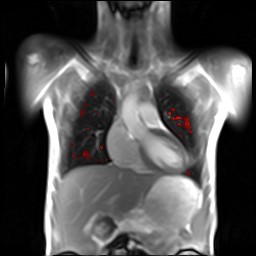

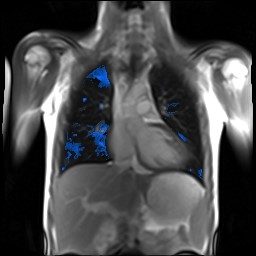

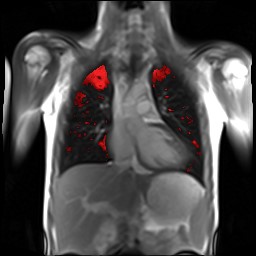

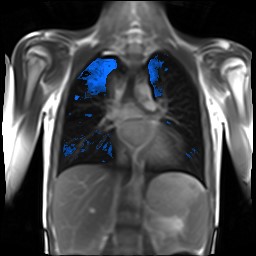

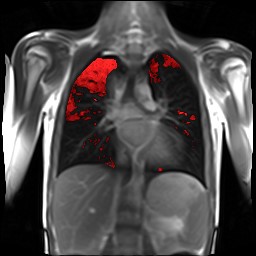

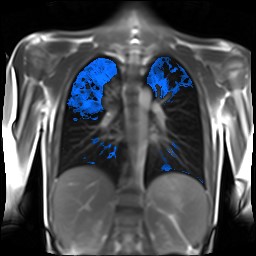

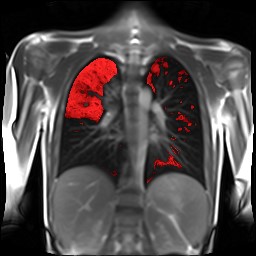

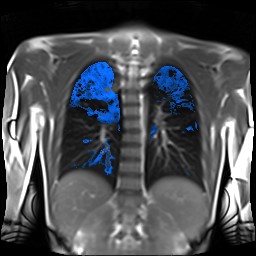

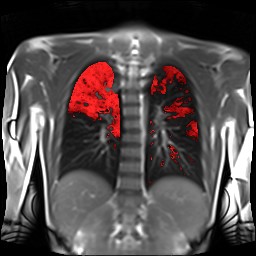

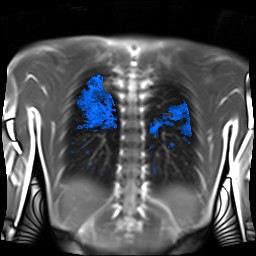

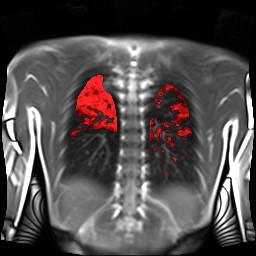


**VQ overlap maps**


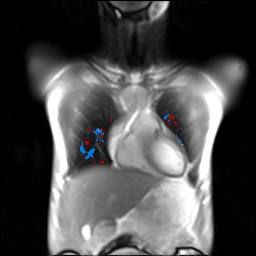

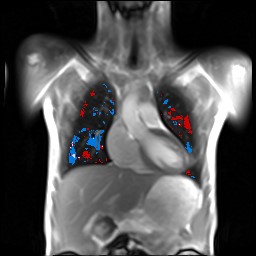

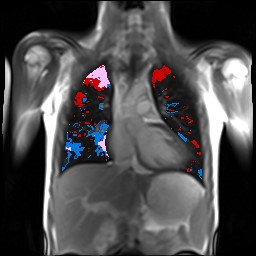

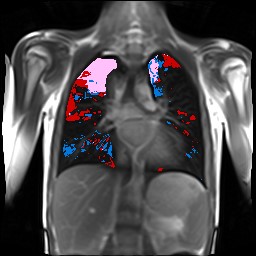

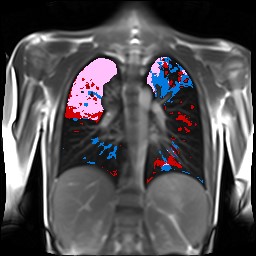

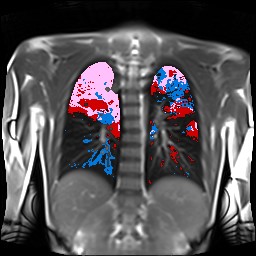


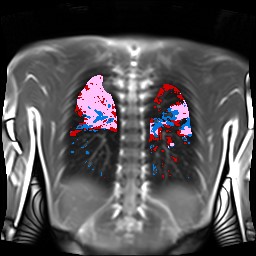


# Legend


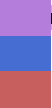
Overlap map colors:

- overlap of V and Q defects (abbreviated as VQO)
- only V defects
- only Q defects
